# Introduction to the Special Issue: Innovations and Strategies for Addressing COVID-19 Pandemic Related Challenges in Prevention Science Research in Applied Settings

**DOI:** 10.1007/s11121-026-01884-5

**Published:** 2026-02-11

**Authors:** Catherine P. Bradshaw, Antonio A. Morgan-López, Rashelle J. Musci

**Affiliations:** 1 University of Virginia, Charlottesville, VA USA; 2 RTI International, Research Triangle Park, Durham, NC USA; 3 Johns Hopkins University, Baltimore, MD USA

**Keywords:** COVID, Measurement, Missing data, Online data collection, Pandemic, Study design

## Abstract

This paper serves as an introduction for a special issue of *Prevention Science*: “Innovations and Strategies for Addressing COVID-19 Pandemic Related Challenges in Prevention Science Research in Applied Settings.” This collection of original papers came together through an open call for original submissions to address emerging issues following the COVID-19 pandemic and related impacts on prevention science research. These papers are organized into four broad themes related to COVID-related impacts on (a) data collection, (b) measurement and missingness, (c) implementation supports and delivery considerations, and (d) broader pandemic impacts. The special issue concludes with a commentary focused on measurement and methodological considerations in analyzing data impacted by COVID-related disruptions. This set of papers provides insights for prevention science scholars and practitioners, illustrates lessons learned for managing pandemic-related data collection and design challenges, and highlights innovations in online data collection.

This special issue of *Prevention Science*—“Innovations and Strategies for Addressing COVID-19 Pandemic Related Challenges in Prevention Science Research in Applied Settings”—features a series of articles focused on the immediate and potential lasting impacts of the COVID-19 pandemic on prevention science research in applied settings, such as communities, schools, homes, and medical settings. This collection of original articles was selected through an open call for letters of intent following a symposium presented during the 2023 Society for Prevention Research conference on this same theme; the panel was designated as an “abstract of distinction” by conference organizers. At the conference, the room was full of prevention research scholars who, like the panel of presenters, had encountered numerous challenges fielding and completing ongoing studies during the pandemic. At that time, it was challenging to forecast the long-term impacts of the pandemic on various aspects of prevention research, including study design, measurement, community engagement, implementation, and trust in research and science. However, it was clear that we as a field were at an inflection point and that we must approach many field-based studies differently from this point forward. Although many communities were expressing an urgent need for increased prevention programming to address growing health disparities and inequities (National Academies of Sciences, Engineering, and Medicine, 2023; Weist et al., 2024) in the USA and worldwide, prevention researchers encountered new and unique implementation barriers.

Prevention scholars were creative, persistent, and resilient, and they did not shy away from the task of adapting what could be adjusted about their approach and design to proceed with the work; this often included a nearly ubiquitous shift to some form of online data collection and adapted modes of program delivery that heavily leveraged the Internet, electronic modules, or were converted to apps. Although there was limited science upon which to base an immediate pivot to online program delivery and data collection, the urgency of the moment necessitated the field’s rapid transformation. Then there was the difficulty of making sense of data collected prior to the onset of the pandemic and those collected during or after the pandemic. This introduced new methodological and measurement challenges such as missing data and changes in participation and response rates and patterns (Carter et al., 2024). Merging pre-pandemic cohorts or data with data collected during or after the pandemic necessitates careful consideration and investigation.

This collection of articles documents some of the innovations introduced, best practices identified, and recommendations resulting from this critical window of time. On both a personal and professional level, this life event seems to have resulted in a lasting shift in the way we design prevention studies in applied settings. Moreover, there has been a change in the way many scholars approach partnership and implementation. For example, many researchers continue to report using more virtual partnership meetings, online recruitment approaches, online assessments, and virtual trainings than prior to the pandemic (Hensen et al., 2021). The use of these techniques has persisted well past the height of the pandemic and does not appear to be declining (Carter et al., 2024). Yet, many field-based researchers have voiced concerns that the experience of the pandemic may have increased burnout, and eroded participant engagement; conversely, remote access provides access to more distant locations and flexible scheduling for researchers, partners, and participants, which may have led to greater resiliency for some partnerships (Donnelly et al., 2021).

A variety of topics were of interest for this special issue, including but not limited to measurement, missing data, study design, implementation science, and research ethics. Not surprisingly, many of the included articles also highlight developmental themes, such as timing issues (e.g., how old an individual was when the pandemic started) and identities/roles in the research study (e.g., child, parent, teacher, provider). The papers also illustrate how the pandemic was a multisystemic stressor, which from an ecological systems perspective had multiple spheres of influence on virtually all aspects of life (e.g., schooling, social, family, workforce, health, economic, sociopolitical, economic, safety). As commentary writer Gottfredson O’Shea (2025) notes, it is helpful to adopt a life course perspective (Elder, 1998) to conceptualize this major life event and consider how it differentially impacted individual trajectories within social and historical contexts.

We have organized the articles within the special issue into four broad cross-cutting themes: data collection, measurement and missingness, implementation supports and delivery considerations, and broader pandemic impacts. This collection of articles provides insights on lessons learned during the pandemic and how best to manage and make the most of data collected during this time. The special issue documents many innovative pivots, such as to online and virtual recruitment, program delivery, implementation support, data collection, participant engagement, and sustainability support. In what follows, we briefly highlight the articles featured in the four sections and some of the main takeaways that are relevant to other prevention science researchers and practitioners. The special issue concludes with an insightful commentary by methodologist Gottfredson O’Shea (2025), who further explores some of the measurement and methodological issues in analyzing data that have been impacted by COVID-related disruptions and discusses implications for future research.

## Special Issue Themes

### COVID-Related Impacts on Data Collection

Despite the growing need for prevention programming during the pandemic, particularly related to behavioral health interventions for children and families, many schools and other applied settings across the USA and around the world closed their doors (Carter et al., 2024). This impacted numerous ongoing research studies being conducted in schools and other applied settings. The first five articles feature examples of studies where researchers quickly pivoted their data collection efforts to online settings to overcome other data collection barriers associated with the pandemic. For example, McLean et al. (2024) describe the procedures for adapting observational data collection for classroom-based video data, which was a common challenge for many ongoing school-based studies during the pandemic. Several studies described how procedures were adapted to reach participants in their homes. For example, Basha et al. (2024) demonstrate that video-recorded parent–child observational data can be feasibly and reliably collected based on findings from a study collected before and during the pandemic. Another example of remote parent and child observations is provided by Tandon et al. (2024), who highlight lessons learned that may be particularly helpful when working with low-income and racially and ethnically diverse parent–child dyads.

Two studies describe overcoming recruitment challenges through social media use. Unger and colleagues (2023) describe the use of alternative approaches to adolescent recruitment during the pandemic, including social media as compared to school-based recruitment. They conclude that recruitment through social media has benefits and may help reach some vulnerable populations for survey research. Tzilos Wernette et al. (2025) describe how they addressed recruitment challenges by shifting from in-person recruitment to remote clinic recruitment through the use of phone, texted messaging, e-mail, and social media campaigns.

### Impact of the Pandemic on Measurement and Missingness

Although there are many potential methodological concerns to be considered, measurement and missing data concerns are particularly salient. The study by Hynes and colleagues (2024) examines data from the COVID-19 Stressors Scale from a national sample of parental caregivers over six time points. Rothenberg and colleagues (2024) undertook the ambitious task of mapping the longitudinal impact of the COVID-19 pandemic on adolescents from 12 cultural groups across the globe. This innovative methodological approach documents the devastating impacts of the pandemic on the lives of youth and serves as an exemplar for modeling the spread and impact of COVID-19 for future public health studies by combining etic and emic approaches to model trajectories.

The paper by Budavari and colleagues (2025) considers various modern missing data methods that may be most relevant in the context of the pandemic. They note a concern about threats to validity when interpreting assessments collected during the pandemic, as measures collected during the pandemic may not reflect the original constructs as intended; these measures may demonstrate measurement noninvariance/differential item functioning. Budavari et al. also highlight potential issues related to measurement and analysis and potential concerns regarding comparing pre- and post-pandemic data and intervention findings.

### Implementation Supports and Delivery Considerations

Several studies document the significant impact of the pandemic on the need to shift the implementation context to accommodate implementation barriers. Shire and colleagues (2024) focus on a study designed to meet the needs of young children with autism that aligns with the Consolidated Framework for Implementation Research model; they describe adaptations to a trial that occurred prior to and following the pandemic and return to in-person classroom services. Fainstein and colleagues (2024) focus on early mathematics learning for kindergarteners during the COVID-19 pandemic using a remote professional development support model. They report findings from a randomized controlled trial results demonstrating favorable outcomes for the remote professional development ROOTS program and how it was helpful in overcoming COVID-19 implementation challenges.

Building on the long line of research on the Family Check-Up, Mauricio and colleagues (2024) adapted the Family Check-Up to address pandemic-related stressors using an online version of the model. Their results demonstrate the promise of the model for supporting caregiver needs and stress and youth depressive symptoms during the pandemic. The study by Park and Lee (2025) illustrates how videoconferencing can be used to deliver the Seoul Premarital Education Program within the context of a quasi-experimental study during the pandemic. They used inverse probability of treatment weighting and found the programming to be a promising approach to prevent marital distress. Finally, the study by Leve and colleagues (2025) focuses on SARS-CoV-2 testing and education supports, comparing partner-located sites to geolocated sites for Latine communities. Leve et al. highlight the utility of propensity score matching to address pandemic-related design challenges.

### Broader Pandemic Impacts

Vásquez-Echeverría et al. (2024) examined over 160,000 5-year-olds’ school readiness scores as rated by preschool teachers in Uruguay from 2020 through 2022 to examine the impact of the pandemic on children’s cognitive, motor, and socioemotional development. They found that scores returned to pre-pandemic levels by 2022, highlighting important implications for future school closures and learning losses. And in their study based in Quebec, Canada, Collet et al. (2026) examined the Resilience Project, which focused on children’s academic and mental health during the pandemic. The authors documented a variety of challenges associated with parental anxiety, depression, and work-life balance during lockdowns and identified several implications for school-based prevention and parental supports.

## Conclusion

In closing, it is our hope that this collection of original empirical articles, and the thoughtful commentary by Gottfredson O’Shea (2025), inspires and motivates scholars to employ some of these innovative solutions to address pandemic-related challenges in prevention science research. Specific guidance is provided regarding how to accurately address and explore for missing data and address design challenges associated with the immediate impact of the COVID-19 pandemic; however, many of these challenges also persist through this post- or “peri-pandemic” period (Weist et al., 2024), as we continue to struggle with recovery, aftermath, and scars left behind (Carter et al., 2024). It is our belief that these articles will help scholars and practitioners to identify innovative methods for addressing challenges encountered in studies fielded during the COVID-19 pandemic. Moreover, many of the implications and lessons learned from these studies could potentially be generalized as important tools for other or future settings, contexts, or historical moments when in-person research activities are hampered. Therefore, this special issue may inform future studies, particularly those using online data collection, programing, or virtual implementation support components for decades to come.
